# Synthetic DNA Delivery of an Optimized and Engineered Monoclonal Antibody Provides Rapid and Prolonged Protection against Experimental Gonococcal Infection

**DOI:** 10.1128/mBio.00242-21

**Published:** 2021-03-16

**Authors:** Elizabeth M. Parzych, Sunita Gulati, Bo Zheng, Mamadou A. Bah, Sarah T. C. Elliott, Jacqueline D. Chu, Nancy Nowak, George W. Reed, Frank J. Beurskens, Janine Schuurman, Peter A. Rice, David B. Weiner, Sanjay Ram

**Affiliations:** a Vaccine & Immunotherapy Center, The Wistar Institute of Anatomy and Biology, Philadelphia, Pennsylvania, USA; b Division of Infectious Diseases and Immunology, University of Massachusetts Medical School, Worcester, Massachusetts, USA; c Genmab, Utrecht, The Netherlands; Albert Einstein College of Medicine

**Keywords:** DNA-delivered monoclonal antibody, *Neisseria gonorrhoeae*, complement, gonorrhea, monoclonal antibodies

## Abstract

Monoclonal antibody (MAb) 2C7 recognizes a lipooligosaccharide epitope expressed by most clinical Neisseria gonorrhoeae isolates and mediates complement-dependent bactericidal activity. We recently showed that a recombinant human IgG1 chimeric variant of MAb 2C7 containing an E430G Fc modification (2C7_E430G), which enhances complement activation, outperformed the parental MAb 2C7 (2C7_WT) *in vivo*. Because natural infection with N. gonorrhoeae often does not elicit protective immunity and reinfections are common, approaches that prolong bacterial control *in vivo* are of great interest. Advances in DNA-based approaches have demonstrated the combined benefit of genetic engineering, formulation optimizations, and facilitated delivery via CELLECTRA-EP technology, which can induce robust *in vivo* expression of protective DNA-encoded monoclonal antibodies (DMAbs) with durable serum activity relative to traditional recombinant MAb therapies. Here, we created optimized 2C7-derived DMAbs encoding the parental Fc (2C7_WT) or complement-enhancing Fc variants (2C7_E430G and 2C7_E345K). 2C7 DMAbs were rapidly generated and detected throughout the 4-month study. While all complement-engaging 2C7 variants facilitated rapid clearance following primary N. gonorrhoeae challenge (day 8 after DMAb administration), the complement-enhancing 2C7_E430G variant demonstrated significantly higher potency against mice rechallenged 65 days after DMAb administration. Passive intravenous transfer of *in vivo*-produced, purified 2C7 DMAbs confirmed the increased potency of the complement-enhancing variants. This study highlights the ability of the DMAb platform to launch the *in vivo* production of antibodies engineered to promote and optimize downstream innate effector mechanisms such as complement-mediated killing, leading to hastened bacterial elimination.

## Abstract (precis)

Neisseria gonorrhoeae has become resistant to most antibiotics in clinical use. Currently, there is no safe and effective vaccine against gonorrhea.

## INTRODUCTION

Transmission of Neisseria gonorrhoeae, the causative agent of the sexually transmitted infection (STI) gonorrhea, occurs through direct mucosal contact with an infected individual that can result in a range of clinical outcomes (1, 2). Infection of the urogenital tract commonly leads to urethritis in men and cervicitis in women, though the rectum and oropharynx are also frequently colonized in both sexes. If not properly treated, infection can lead to serious complications, including pelvic inflammatory disease (PID), ectopic pregnancy, infertility, chronic pelvic and/or abdominal pain, and an increased risk of contracting HIV (3, 4). In rare cases, dissemination from mucosal sites can result in systemic dermatitis, arthritis, tenosynovitis, meningitis, or endocarditis (5). By some estimates, nearly half of all N. gonorrhoeae cases are asymptomatic, allowing continuous communal transmission via asymptomatic carriers (6–9). Concerningly, primary infection does not appear to induce protective immunity but, rather, may increase susceptibility to subsequent infections in the months following initial clearance (7, 10–16). Asymptomatic transmission combined with the lack of protective immunity following natural infection contributes to the ongoing epidemic. The past decade has seen a sustained, steady increase in the number of N. gonorrhoeae cases in the United States; in 2018, 583,405 reported cases occurred the United States, a 63% increase since 2014 (Centers for Disease Control and Prevention [CDC]) (17).

As there are no prophylactic agents available to prevent gonococcal transmission, current control efforts rely on the use of antibiotics for treatment of identified cases. Unfortunately, this approach may not be sustainable, as N. gonorrhoeae has developed resistance to almost every antibiotic in clinical use (18–20). Alarmingly, resistance to ceftriaxone and azithromycin, which had been the first-line treatments for gonorrhea recommended by the CDC, has been reported from several countries (19–22). As a result, azithromycin is no longer recommended; ceftriaxone alone at a higher dose is recommended by the CDC for the treatment of uncomplicated gonorrhea. As the number of gonorrhea cases continues to rise worldwide, additional tools to combat the global spread of multidrug-resistant gonorrhea are urgently needed. Antibody-based therapeutics are a promising approach to both prevent and treat a number of infectious diseases (23–25). Monoclonal antibody (MAb) 2C7 targets a highly conserved glycan epitope in gonococcal lipooligosaccharide (LOS) which is expressed by over 95% of all isolates examined directly *ex vivo* or following limited passages *in vitro* (26–28). The loss of the 2C7 epitope significantly compromises gonococcal colonization of mouse vaginas in an experimental model, indicating a potentially important role in pathogenesis (28–30). Accordingly, MAb 2C7 IgG exhibited bactericidal activity against all 62 minimally passaged gonococcal isolates tested from Nanjing, China (28). The *in vivo* efficacy of recombinant MAb 2C7 IgG against N. gonorrhoeae challenge was studied and validated using a mouse vaginal colonization model; activity of MAb 2C7 required an intact complement pathway (31). The potency of 2C7 was improved by introducing an E-to-G mutation in the Fc of human IgG1 (31); Fc mutations such as E430G and E345K promote Fc-Fc interactions that lead to increased hexamer formation, C1q engagement, and complement activation (32, 33).

The bactericidal activity and efficacy *in vivo* of MAb 2C7_E430G make it a promising candidate for further development. However, treatment regimens using recombinant MAbs produced with traditional bioprocessing approaches are costly, often requiring repeated dosing due to the short serum half-life of recombinant IgG. As reinfection with N. gonorrhoeae is common, particularly within the first 6 to 12 months following primary infection (7, 10–16), an ideal immunotherapeutic would both clear the primary infection and demonstrate prolonged *in vivo* activity to prevent or control secondary infection during this window of increased vulnerability. *In vivo* delivery of synthetic nucleic acid expression vectors encoding engineered MAb genes represents a novel approach to circumvent the challenges associated with production, delivery, and longevity of traditional MAb therapy (34).

Improvements in the design and genetic engineering of DNA-encoded monoclonal antibodies (DMAbs) have yielded biologics with improved expression and *in vivo* activity in numerous models of infectious diseases, including those caused by Ebola virus (35), influenza A and B viruses (36), drug-resistant Pseudomonas aeruginosa (37), Zika virus (38), HIV (39), and Lyme disease (40). Approaches to further improve the *in vivo* efficacy of DMAb-based therapeutics are under investigation, including those that enhance molecular potency by improving their innate effector functions. Here, this advanced DMAb technology was evaluated to develop and study the *in vivo* delivery of modified 2C7-based antibody variants with improved capacity to induce complement-mediated killing relative to the wild-type antibody, resulting in an immunotherapeutic with potent and extended *in vivo* bactericidal activity against experimental N. gonorrhoeae infection.

## RESULTS

### Engineering and expression of 2C7-based DMAbs containing Fc modifications to enhance downstream complement activation.

Using a designed dual plasmid approach, V_H_ and V_L_ sequences for MAb 2C7, along with those for constant domains of human IgG1, were genetically optimized at the nucleic acid (DNA and RNA) levels and inserted into customized pVax DNA plasmid DNA expression vectors under the control of a human cytomegalovirus (hCMV) promoter, optimized IgG leader sequences, and a bovine growth hormone (BGH) poly(A) signal (Fig. S1A) as previously described (35). In addition to those expressing the light-chain (LC_WT) and heavy-chain (HC_WT) 2C7 wild-type sequences, three additional heavy-chain plasmids were generated in order to assess effect of complement-modulating Fc modifications on the *in vivo* bactericidal activity of 2C7-based antibodies. These additional 2C7 HC variants included two enhancing variants, HC_E430G and HC_E345K; a recombinant human IgG1 chimeric variant of MAb 2C7 bearing the E430G Fc mutation enhanced complement activation and accelerated clearance of gonococci in the mouse vaginal colonization model (31). The third variant, called HC_K322A/D270A (abbreviated as 2C7_A/A), is a double mutant in which complement activation is abrogated. The HC plasmids for each 2C7 variant, combined with the common LC plasmid, were cotransfected *in vitro* (Fig. S1A). Coexpression of these optimized plasmid combinations resulted in the assembly and secretion of 2C7 DMAbs into the culture supernatants, as demonstrated via Western blot analysis (Fig. S1B).

10.1128/mBio.00242-21.1 FIG S1 Dual-plasmid design and *in vitro* expression of 2C7 DMAb variants. (A) Schematic of synthetic-DNA dual plasmids. The 2C7 light chain (LC) is composed of the variable light (V_L_; white) sequence followed by the constant λ light chain of human IgG1. 2C7 heavy chain (HC) plasmids were generated in which the variable heavy (V_H_; dark gray) sequence is followed by the constant heavy (C_H_) of human IgG. The four 2C7 variants included the (i) unmodified hIgG1 HC (2C7_WT; black), (ii) D270A/K322A double mutant (2C7_A/A; bright pink), (iii) E345K single mutant (2C7_E345K; blue), and (iv) E430G single mutant (2C7_E430G; green). All HC and LC sequences were optimized for *in vivo* expression and engineered using a human cytomegalovirus (hCMV) promoter, optimized IgG leader sequences (light pink), and a bovine grown hormone (BGH) poly(A) signal inserted into a pVax expression vector. (B) *In vitro* expression of 2C7 DMAb variants. Western blot analysis of culture supernatant following *in vitro* transfection of the indicated plasmid combinations. 2C7 DMAbs were detected with anti-human IgG (H+L) (2C7 DMAbs; green). Anti-GAPDH is shown for loading (red). Download FIG S1, TIF file, 0.4 MB. Copyright © 2021 Parzych et al. 2021 Parzych et al. https://creativecommons.org/licenses/by/4.0/ This content is distributed under the terms of the Creative Commons Attribution 4.0 International license.

### Facilitated delivery of 2C7-based DMAbs results in rapid, robust, and sustained *in vivo* expression.

**FIG 1:**
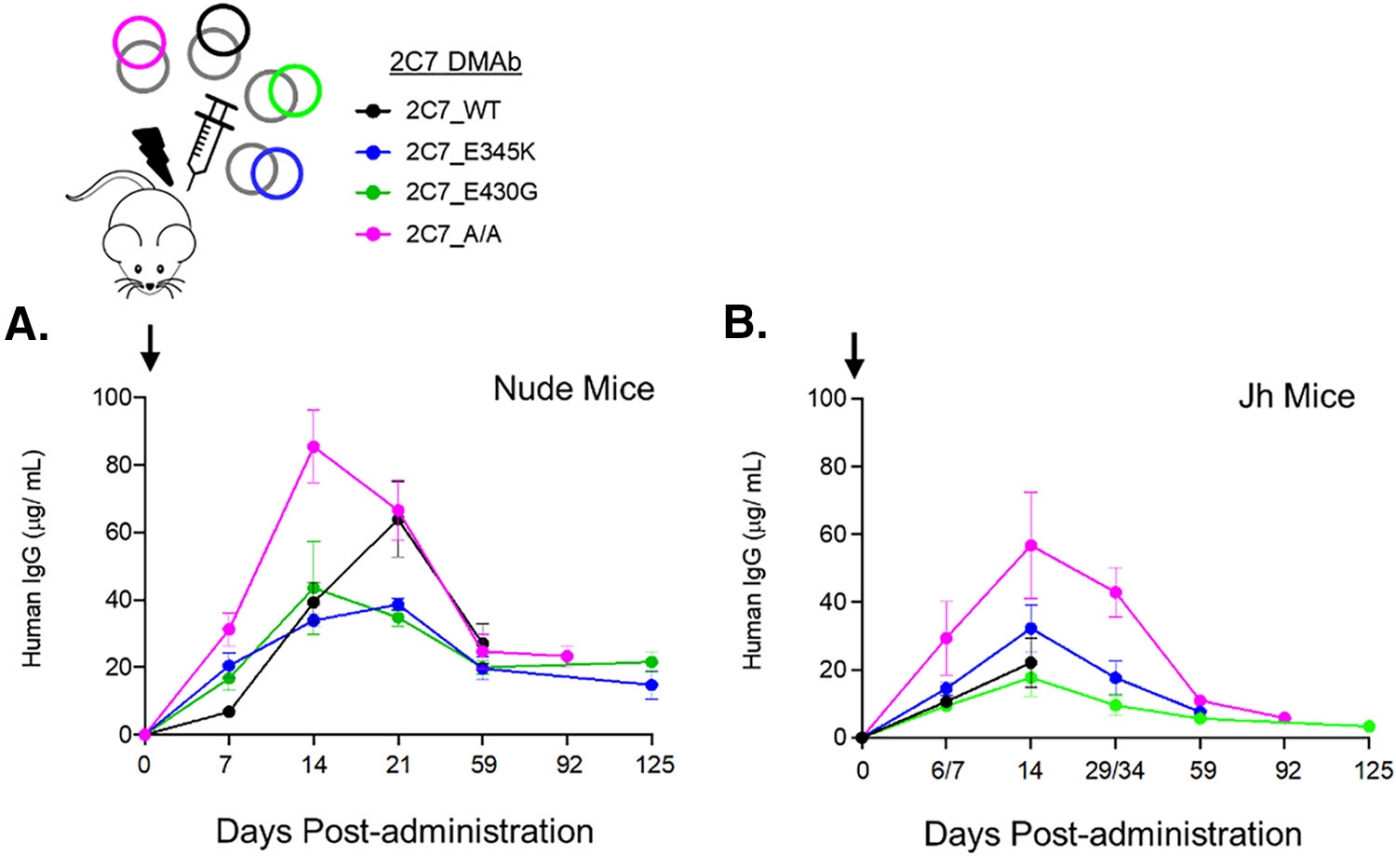
Rapid, robust, and durable *in vivo* expression of 2C7 DMAb variants. Groups of (A) nude mice (*n* = 3) and (B) Jh mice (*n* = 3) were injected intramuscularly with the dual-plasmid systems encoding the indicated 2C7 DMAbs, facilitated by CELLECTRA-EP (D0; black arrows). Serum levels of chimeric 2C7 DMAbs were measured over time by ELISA using plates coated with anti-human IgG. Bound antibodies were detected with anti-human IgG–HRP and quantified against a purified hIgG standard curve. Graphs depict the average group concentrations (± standard errors of the means [SEM]) of each 2C7 variant in the sera at the indicated time point after DMAb administration.

To evaluate the kinetics and durability of *in vivo* expression, mammalian expression plasmid DNA carrying cDNA of antibody heavy and light chains of 2C7 variants was administered to groups of mice via intramuscular (i.m.) injection followed by CELLECTRA-3P electroporation (EP). Expression of 2C7 variants induced by plasmid DNA constructs delivered by i.m. EP was initially assessed in two immunodeficient mouse models (Jh mice and nude mice) that lack the ability to mount adaptive antibody responses against the human CH1, CH2, CH3, and CL domains of these variants. Quantification of anti-human IgG in the sera of mice given DMAb demonstrated the rapid and robust expression of all 2C7 DMAbs in both nude (Fig. 1A) and Jh (Fig. 1B) strains. All six mice receiving DMAb seroconverted by D7 postadministration, and the majority (63%) of mice had serum levels of human IgG exceeding 10 μg/ml at that time. Serum concentrations increased thereafter, achieving peak levels 14 to 21 days postadministration that exceeded >20 μg/ml in 75% of animals. These levels gradually contracted to approximately half of peak titers by day 59 (D59), at which point all 2C7 variants were detected at lower but significant levels (>4 μg/ml). Thereafter, 2C7 DMAb levels remained stable through D92 for all variants. These studies demonstrated facilitated DNA delivery that resulted in rapid and durable *in vivo* production, assembly, and secretion of 2C7-based DMAbs containing different Fc modifications designed to modulate complement engagement.

### *In vivo*-produced 2C7 DMAbs recognize the LOS antigen and exhibit complement-dependent bactericidal activity.

**FIG 2:**
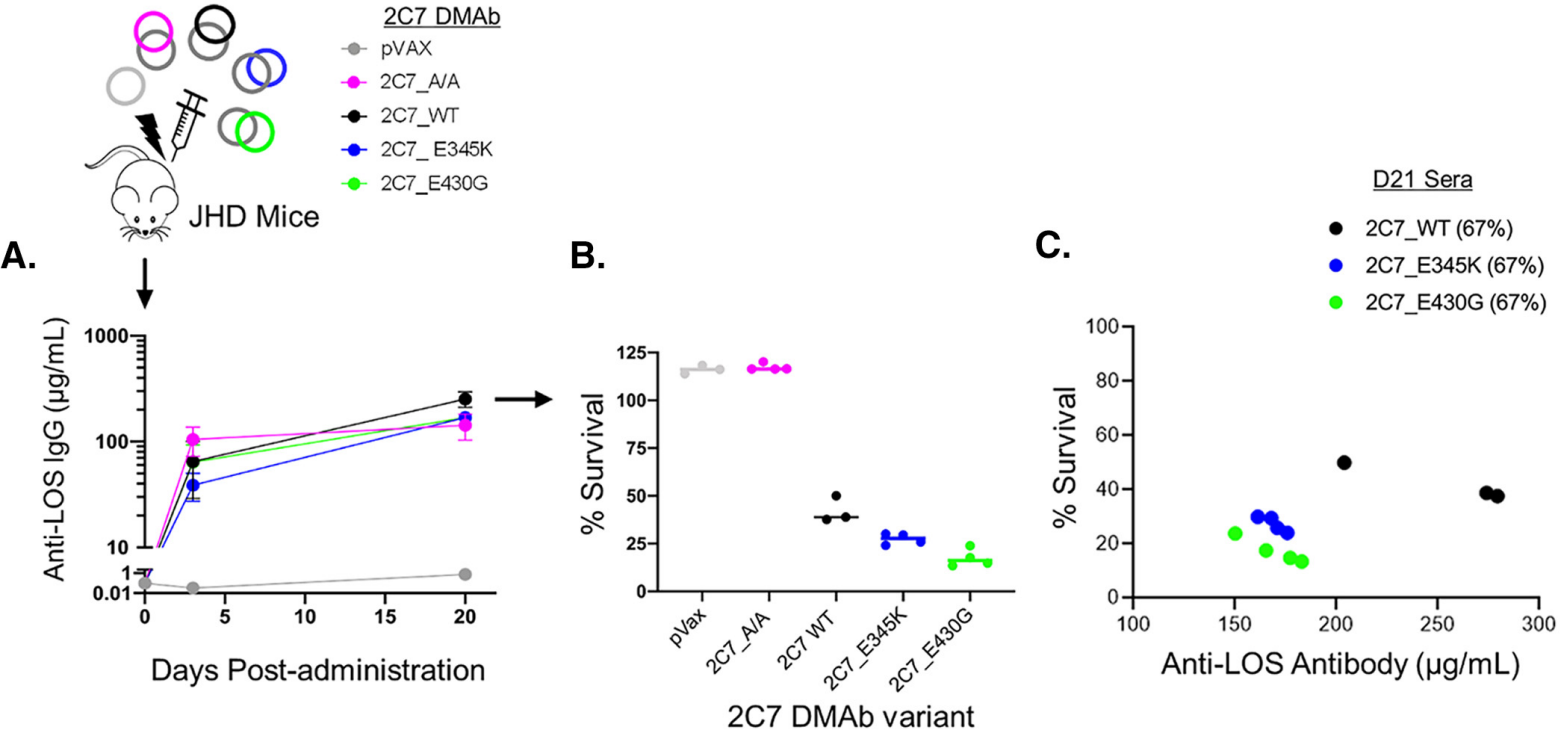
*In vivo*-produced 2C7 DMAbs bind N. gonorrhoeae LOS and are bactericidal. (A) JHD mice (*n* = 3 or 4/group) were given the indicated 2C7 DMAbs (D0; black arrow) or pVax vector as a control. Anti-LOS titers in sera at D3 and D20 were measured by ELISA using LOS purified from N. gonorrhoeae strain 15253. Bound antibodies were detected with anti-human IgG-AP and quantified against a standard curve using purified recombinant 2C7 MAb. The graph depicts the average group concentrations (± SEM) of each 2C7 DMAb in mouse sera at the indicated time point. (B) Function of the 2C7-based DMAbs was assessed *in vitro*. Bacterial survival (CFU) in the presence of the indicated D20 serum samples (67% [vol/vol]) and human complement (16.7% [vol/vol]) was determined using a serum bactericidal assay. The graph depicts the percent survival for each D20 sample at 30 min of incubation relative to 0 min; horizontal lines indicate group averages. (C) Correlation of serum anti-LOS titer and bacterial survival of each D20 serum sample from 2C7_WT-, 2C7_E345K-, and 2C7_E430G-immunized mice.

The specificity and functionality of *in vivo*-produced 2C7 DNA-encoded monoclonal antibodies were evaluated *in vitro*. 2C7 DMAb plasmids were administered to immunocompromised JHD mice, the antibody-deficient strain selected to assess *in vivo* efficacy in a mouse vaginal colonization model of N. gonorrhoeae. As shown in Fig. 2A, DMAb expression derived from electroporation of DMAb plasmids into muscle was detected in serum as early as 72 h after plasmid delivery, at which point 100% of mice produced antigen-specific 2C7 IgG that bound specifically to LOS by ELISA. Levels of each 2C7 variant further increased by D20, resulting in high and similar titers in sera (Fig. 2A). DMAb function was then assessed using an *in vitro* bactericidal assay where sera from DMAb-administered mice were incubated with N. gonorrhoeae strain FA1090 in the presence of human complement. As expected, sera containing complement-engaging variants (2C7_WT, 2C7_E345K, and 2C7_E430G) all possessed significant bactericidal activity (≤50% survival) (Fig. 2B) compared to the complement-null variant (2C7_A/A), which did not facilitate bacterial killing despite expression and binding to LOS similar to those of the complement-active 2C7 variants (Fig. 2A). Importantly, the sera containing 2C7_E430G appeared to possess higher killing activity (i.e., lower survival) than those with 2C7_WT, despite somewhat lower serum concentrations, reiterating the increased potency of this complement-enhancing variant (Fig. 2C). Similar LOS binding and superior bactericidal activity of the 2C7_E430G variant were noted in D3 and D21 sera harvested from Jh mice that had received 2C7 DMAbs (Fig. S2). Importantly, 2C7 DMAbs were also detected in the vaginal cavity at D20 postadministration (Fig. S3). Hence, DNA delivery of optimized 2C7 plasmids results in the *in vivo* production and systemic distribution of engineered and functional 2C7 MAbs.

10.1128/mBio.00242-21.2 FIG S2 Bactericidal activity of 2C7 DMAb variants expressed in Jh mice. (A) *In vitro* functionality of 2C7-DMAb sera (Jh mice; 3 or 4/group). N. gonorrhoeae strain FA1090 was incubated with D3 (left) or D21 (right) sera of mice given DMAb at a final concentration of 33% (vol/vol) (circles/open bars) or 67% (vol/vol) (squares/hatched bars) in the presence of human complement (∼16.7% [vol/vol]). The graph depicts the percent bacterial survival afforded by each serum sample as well as the group averages (± standard deviation [SD]) relative to control plates. (B) Correlation of anti-LOS titers versus bactericidal activity. Anti-LOS titers in the same D3 (left) and D21 (right) serum samples were determined via ELISA using plates coated with purified LOS antigen (N. gonorrhoeae strain 15253). Bound antibodies were detected with anti-human IgG-AP and quantified against a standard curve using purified recombinant 2C7 MAb. The graph depicts the 2C7 concentrations (*x* axis) and bactericidal activity (*y* axis) for individual serum samples, which are colored according to DMAb group. These parameters were analyzed using a Spearman correlation (*r*, correlation coefficient). Download FIG S2, TIF file, 0.3 MB. Copyright © 2021 Parzych et al. 2021 Parzych et al. https://creativecommons.org/licenses/by/4.0/ This content is distributed under the terms of the Creative Commons Attribution 4.0 International license.

10.1128/mBio.00242-21.3 FIG S3 Detection of 2C7 DMAbs in mouse vaginal secretions. JHD mice (*n* = 3 or 4/group; from the group of mice used for Fig. 2) were given the indicated 2C7 DMAb plasmids. At D20, vaginas were swabbed, and swabs were eluted in 100 μl saline. Anti-LOS IgG was measured by ELISA using purified LOS from N. gonorrhoeae strain 15253. Bound antibodies were detected with anti-human IgG-AP and quantified against a standard curve using purified recombinant 2C7 MAb. Graphs depict the individual 2C7 DMAb concentrations found in washes; the horizontal bars represent group averages. Download FIG S3, TIF file, 0.09 MB. Copyright © 2021 Parzych et al. 2021 Parzych et al. https://creativecommons.org/licenses/by/4.0/ This content is distributed under the terms of the Creative Commons Attribution 4.0 International license.

### Complement-engaging 2C7 DMAbs provide both early and durable efficacy against N. gonorrhoeae challenge.

**FIG 3:**
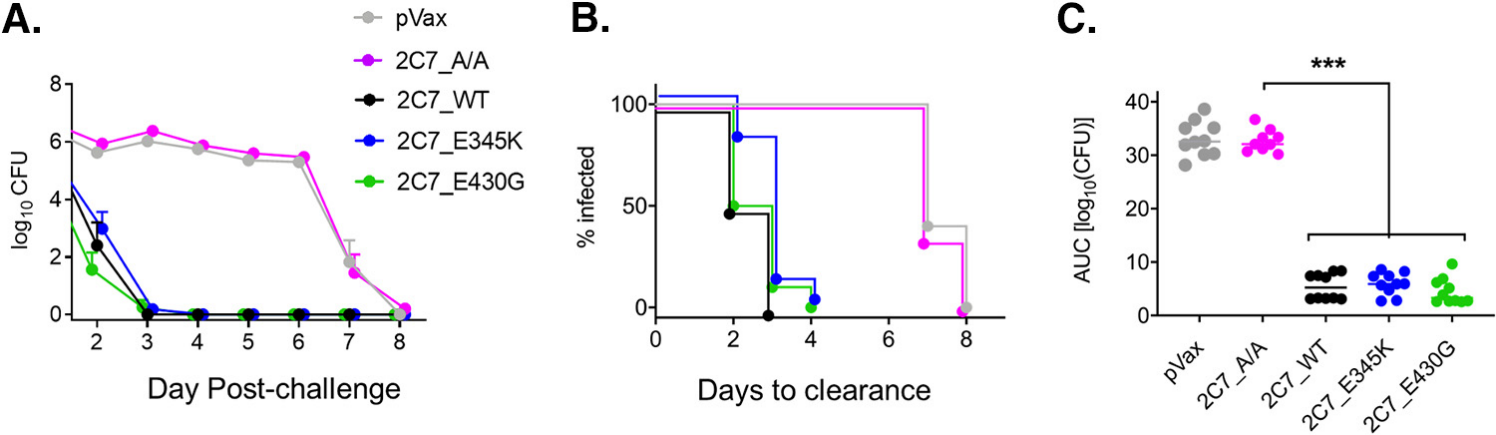
Complement-activating 2C7 DMAb variants effectively clear primary colonization in the N. gonorrhoeae vaginal colonization model. Groups of JHD mice (9 or 10/group) were administered the indicated 2C7 variants (D0) and challenged (D8) with N. gonorrhoeae FA1090 (4.2 × 10^7^ CFU) intravaginally (see Fig. S4 for experimental design). Infection was monitored by vaginal swabbing. (A) Bacterial burden following primary infection. The graph depicts the average log_10_ CFU (mean ± SEM) detected in vaginal secretions on the indicated days post-challenge. (B) Time to bacterial clearance is shown using Kaplan-Meier curves, which display the percentage of each group with detectable vaginal CFU on the given day post-challenge. (C) Overall bacterial burden (each point represents a single mouse) was assessed using AUC (log_10_ CFU) analysis. AUC values for each group were compared using the Kruskal-Wallis test followed by Dunn’s *post hoc* test (***, *P < *0.0001).

**FIG 4:**
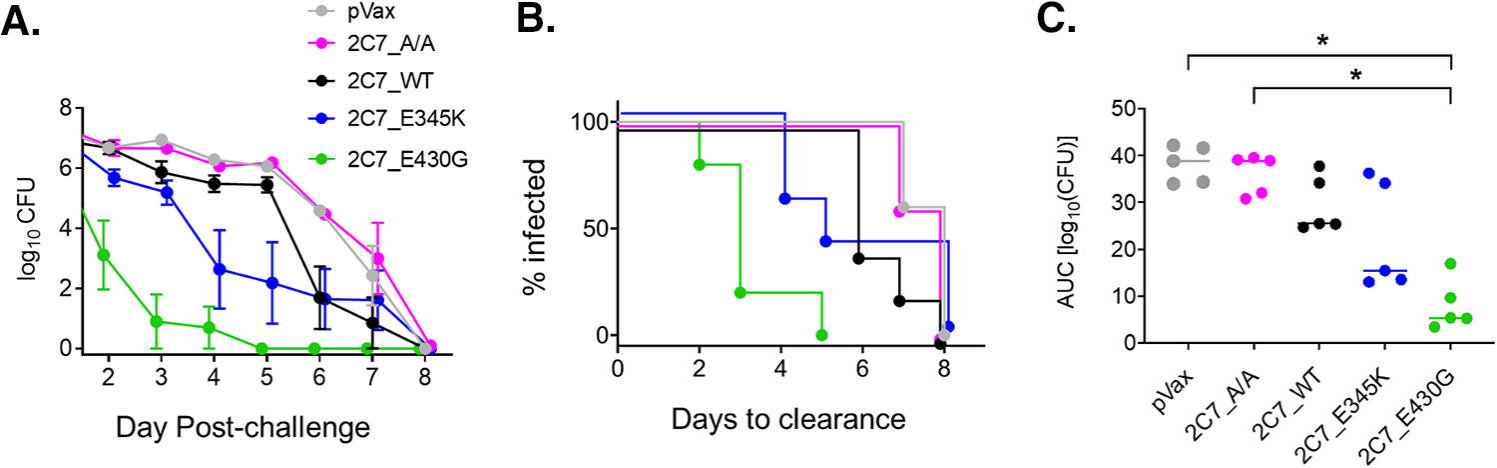
2C7_E430G DMAb demonstrates durable protection following delayed rechallenge with N. gonorrhoeae. Mice that were initially challenged and were in the diestrus phase of the estrous cycle and thus amenable to reinfection (*n* = 5/group) underwent a rechallenge on D65 postadministration (see Fig. S4 for experimental design) with N. gonorrhoeae FA1090 (3.5 × 10^7^ CFU). Bacterial burden was monitored daily by vaginal swabbing. (A) Bacterial burden following rechallenge. The graph depicts the average log_10_ CFU (mean ± SEM) detected in the vaginal mucosa on the indicated days postinfection. Comparison across groups using a mixed-effects model with a cubic fit showed significant differences between 2C7_E430G and each of the other groups (*P* < 0.001 versus pVax, 2C7_A/A, and 2C7_WT; *P* = 0.0017 versus 2C7_E435K). (B) Time to bacterial clearance is shown using Kaplan-Meier curves, which display the percentage of each group with detectable vaginal CFU on the given day post-challenge. (C) Estimated bacterial burden over time (cumulative infection) computed for each mouse using AUC (log_10_ CFU) analysis. Each point represents an individual mouse. AUC values for each group were compared using the Kruskal-Wallis test followed by Dunn’s *post hoc* test (*, *P* < 0.05).

Previous reports have studied the ability of the recombinant chimeric 2C7 MAb to facilitate clearance of infection using a vaginal colonization model of N. gonorrhoeae (31). Here, we utilized this model to assess the efficacy of 2C7-based DMAbs to hasten bacterial clearance following early (D8 postadministration) (Fig. 3) and delayed (D65 postadministration) (Fig. 4) challenge (see Fig. S4 for the experimental design). Plasmid 2C7 DMAb variants were delivered to JHD mice via i.m. EP, and subsequent DMAb expression in mouse sera was verified on D3 postadministration (Fig. S5). Mice were then challenged intravaginally with N. gonorrhoeae strain FA1090 on D8 after DMAb delivery, and bacterial loads were monitored daily. All groups expressing complement-engaging 2C7 DMAbs (2C7_WT, 2C7_E430G, and 2C7_E345K) demonstrated similar and rapid bacterial control, represented by a sharp decline in vaginal CFU (Fig. 3A) and clearance of infection within 4 days (median times to clearance for 2C7_WT, 2C7_E430G, and 2C7_E345K were 2.5, 2.5, and 3 days, respectively) (Fig. 3B). This was markedly faster than the median time to clearance for the control groups (empty pVax vector and the complement-null 2C7_A/A mutant), which ranged from 7 to 7.5 days. The overall burden of infection, measured by analysis of the area under the curve, was similarly and significantly reduced in the groups that expressed complement-active 2C7 DMAbs relative to control groups (*P < *0.0001) (Fig. 3C). These data show that prophylactic delivery of highly expressing, complement-activating 2C7 DMAbs accelerates clearance of N. gonorrhoeae colonization soon after immunization.

10.1128/mBio.00242-21.4 FIG S4 Designs of 2C7 DMAb efficacy study using the N. gonorrhoeae vaginal colonization model in JHD mice. (A) DMAb administration. Groups of JHD mice (9 or 10/group) were given the indicated 2C7 DMAb plasmids (or pVax control plasmid). All mice were bled throughout the experiment (red drops) to monitor 2C7 expression kinetics (Fig. 4). (B) Primary challenge. On D8 post-DMAb administration, mice were challenged with N. gonorrhoeae (FA1090 strain; 4.2 × 10^7^ CFU, intravaginally), and infection burden was monitored daily (enumeration of vaginal CFU). Bacterial control (daily CFU) and time to clearance were calculated for each treatment group (Fig. 3). (C) Secondary challenge (rechallenge). On D65 postadministration, mice that had had undergone primary challenge and were in the diestrus phase of the estrus cycle and therefore could be infected with N. gonorrhoeae (5/group) were rechallenged with N. gonorrhoeae (FA1090 strain; 3.5 × 10^7^ CFU, intravaginally). Infection was monitored as described above. Download FIG S4, TIF file, 0.2 MB. Copyright © 2021 Parzych et al. 2021 Parzych et al. https://creativecommons.org/licenses/by/4.0/ This content is distributed under the terms of the Creative Commons Attribution 4.0 International license.

10.1128/mBio.00242-21.5 FIG S5 Serum DMAb expression levels over time in N. gonorrhoeae-challenged JHD mice. JHD mice (9 or 10/group) were given the indicated 2C7 DMAbs on day 0 (vertical arrows) (Fig. S4). Anti-LOS DMAb levels in sera were measured at the indicated time points postadministration by ELISA against LOS purified from gonococcal strain 15253. IgG bound to LOS was detected with anti-human IgG-AP and quantified against a standard curve (purified recombinant 2C7 MAb). The graphs depict individual titers in challenged mice; levels in mice that were rechallenged at D65 are indicated by open circles. The horizontal lines represent the mean 2C7 concentrations of the group. As a point of reference, 1 μg/ml is indicated by the red dashed line. Download FIG S5, TIF file, 0.4 MB. Copyright © 2021 Parzych et al. 2021 Parzych et al. https://creativecommons.org/licenses/by/4.0/ This content is distributed under the terms of the Creative Commons Attribution 4.0 International license.

To better define the kinetics of *in vivo* expression and functional durability each 2C7 DMAb variant, mice were monitored for >2 months post-challenge. Robust expression levels (>20 μg/ml) were achieved in all mice given 2C7, which remained high for several weeks (through D70) before decreasing by D85 (Fig. S5). Even at this delayed time point, mean 2C7 IgG antibody levels for each DMAb group exceeded 5 μg/ml. To evaluate the relative long-term efficacy of 2C7 DMAbs, mice that had undergone primary challenge and were in the diestrus phase of the estrous cycle and therefore amenable to recolonization were rechallenged with strain FA1090 on D65. While parental 2C7_WT had a modest effect on infection kinetics (Fig. 4A and B), 2C7_E430G was particularly potent, clearing secondary infection faster than all other groups (median time to clearance, 3 days, versus 5, 6, and 8 days for the 2C7_E345K, 2C7_WT, and 2C7_A/A groups, respectively) (Fig. 4B). Mice expressing the 2C7_E430G DMAb had significantly lower overall bacterial burdens than control mice (*P < *0.05) (Fig. 4C). Collectively, these studies demonstrate superior and extended activity of the 2C7_E430G variant against delayed secondary infection.

### Passive transfer of purified 2C7 DMAbs confirms superior protection afforded by the 2C7_E430G variant at dose-sparing levels.

**FIG 5:**
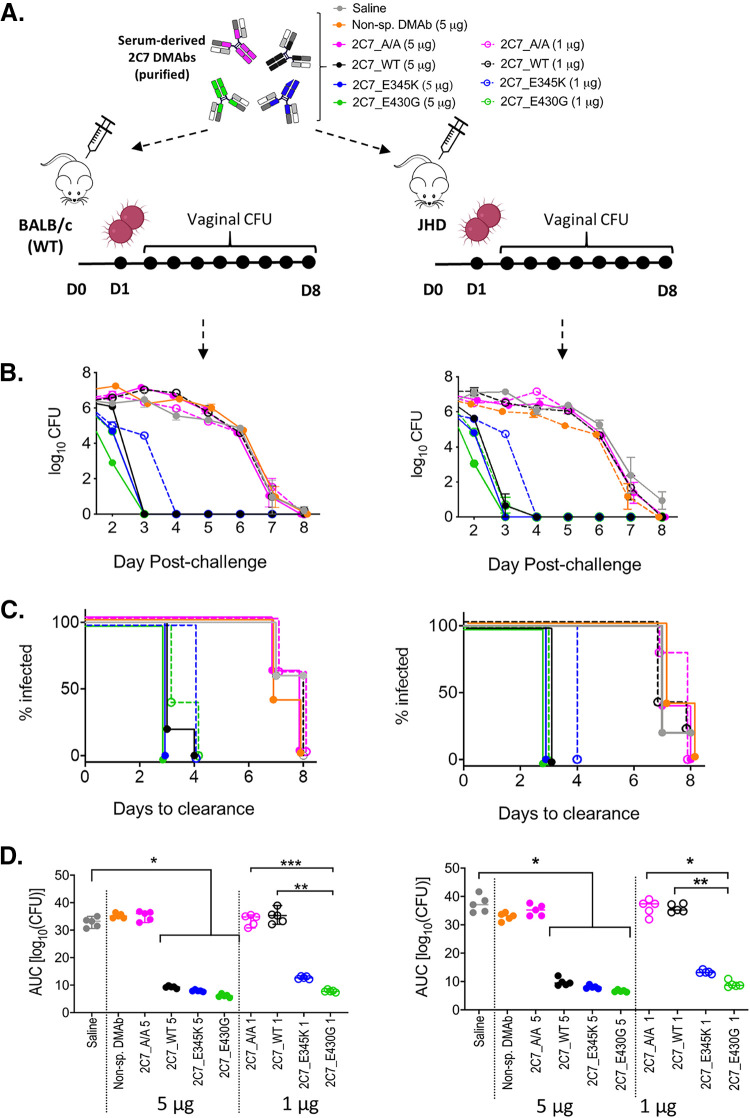
Passive transfer studies verified the superior efficacy of 2C7 _E340G. (A) Experimental layout of the passive transfer study. The indicated 2C7 DMAbs were purified from sera pooled from immunized JHD mice. On D0, groups (*n* = 5) of naive BALB/c mice (left) and JHD mice (right) received intravenous injections of purified 2C7 DMAb variants at a single dose of 5 μg (solid circles) or 1 μg (open circles). Mice were challenged intravaginally with N. gonorrhoeae FA1090 (3.2 × 10^7^ CF) a day later on D1. (B) Average bacterial burden (log_10_ CFU [mean ± SEM]) measured daily in vaginal secretions after infectious challenge on D1. (C) Time to bacterial clearance is shown using Kaplan-Meier curves, which display the percentage of each group with detectable vaginal CFU, measured daily after infectious challenge on D1. (D) Overall bacterial control in each animal was assessed using AUC (log_10_ CFU) analysis. AUC values for each group were compared using the Kruskal-Wallis test followed by Dunn’s *post hoc* test (*, *P* < 0.05; **, *P < *. 001; ***, *P < *0.0001).

Variation in DMAb serum concentrations across groups confounded the ability to evaluate relative efficacy afforded by different complement-enhancing Fc mutations. To control for expression levels and determine the relative efficacies *in vivo* of these constructs, we purified DMAbs from sera of JHD mice and tested the potency of each 2C7 variant when it was passively transferred into wild-type BALB/c and JHD mice at defined doses (5 μg or 1 μg, delivered intravenously [i.v.] as a single dose) (Fig. 5A). As a nonspecific DMAb control, an additional group received a purified, previously characterized anti-HIV DMAb HIV (39). Mice were challenged intravaginally on the following day with N. gonorrhoeae FA1090. All complement-activating DMAbs (2C7_WT, 2C7_E345K, and 2C7_E430G), when administered intravenously at a single dose of 5 μg, rapidly reduced bacterial loads (Fig. 5B), leading to complete clearance within 3 to 4 days (Fig. 5C). Clearance in these groups was significantly faster than in the 3 control groups (2C7_A/A, nonspecific DMAb, and pVax controls), where infection did not clear until 8 days (*P < *0.05). Comparison of values of area under the curve (AUC) for the complement-engaging variants demonstrated superior activity of the 2C7_E430G variant relative to the parental 2C7_WT DMAb at the 5-μg i.v. dose in both strains of mouse (*P < *0.05) (Fig. 5D). At the 5-μg i.v. dose, 2C7_E345K treatment appeared intermediate (i.e., between 2C7_WT and 2C7_E430G) in the AUC analysis; however, it cleared infection within the same time frame as 2C7_E430G (3 days) (Fig. 5B and C). These findings were consistent in both mouse strains.

Differences in the relative potency of the purified 2C7 DMAb variants were further revealed when the 1-μg i.v. dose of DMAb 2C7_WT was shown to be ineffective in clearing infection (Fig. 5B to D). Conversely, 2C7_E430G retained potency at this low dose, indicated by faster median times to clearance than in control groups (3 days versus 7 to 8 days) (Fig. 5B and C). AUC analysis also showed lower bacterial burdens achieved by the 1-μg i.v. dose of 2C7_E430G relative to the parental 2C7_WT and control groups (Fig. 5D). These data confirm the enhanced potency of DNA-delivered, *in vivo*-produced 2C7_E430G compared to 2C7_WT when used at similar concentrations. Finally, we demonstrated a correlation between MAb levels and efficacy using recombinant chimeric 2C7_E430G, where clearance of colonization occurred in a dose-responsive manner (Fig. S6). This experiment reiterates the importance of attaining a high concentration of MAb 2C7, which is achieved by the DMAb platform.

10.1128/mBio.00242-21.6 FIG S6 *In vivo* dose response of chimeric 2C7_E430G recombinant IgG (rIgG). Purified 2C7_E430G IgG was produced in CHO cells using traditional bioprocessing strategies and administered systemically at the indicated dose (1, 5, or 25 μg/animal; *n* = 5/group) on D0. The following day, mice were challenged intravaginally with N. gonorrhoeae (D1) and infection was monitored via daily vaginal swabbing (Fig. 5A). (A) Bacterial burden following challenge. Graphs depict the average log_10_ CFU (mean ± SEM) detected in the vaginal mucosa on the indicated days postinfection. (B) Time to bacterial clearance is shown using Kaplan-Meier curves, which display the percentage of each group with detectable vaginal CFU on the given day post-challenge. Pairwise comparisons were made with the Mantel-Cox log-rank test, and significance was set at 0.008 (Bonferroni’s correction for 4 groups). The following comparisons were significant: saline versus 2C7_E430G (1 μg), *P = *0.003; saline versus 2C7_E430G (5 μg), *P = *0.002; saline versus 2C7_E430G (25 μg), *P = *0.002; and 2C7_E430G (1 μg) versus 2C7_E430G (25 μg), *P* = 0.002. (C) Overall bacterial control in each animal was assessed using AUC (log_10_ CFU) analysis. AUC values for each group were compared using the Kruskal-Wallis test followed by Dunn’s *post hoc* test (*, *P* < 0.05; ***, *P < *0.0001; ns, not significant). Download FIG S6, TIF file, 0.1 MB. Copyright © 2021 Parzych et al. 2021 Parzych et al. https://creativecommons.org/licenses/by/4.0/ This content is distributed under the terms of the Creative Commons Attribution 4.0 International license.

## DISCUSSION

A steady increase in incidence combined with growing rates of drug resistance has led the CDC to classify N. gonorrhoeae as an “urgent health threat” according to a 2019 antibiotic resistance threat report (41). In 2018, nearly half of all clinical N. gonorrhoeae isolates showed resistance to at least one antibiotic. A safe and effective vaccine that confers durable protection against gonorrhea would be highly desirable but has proven elusive thus far. N. gonorrhoeae has evolved numerous strategies to subvert protective host immune responses (20, 42). Thus, it is not surprising that natural infection does not confer protective immunity and reinfections are relatively common (7, 10–16). Recently, administration of doxycycline as either pre- or postexposure prophylaxis to prevent bacterial STIs among high-risk populations of men who have sex with men (MSM) has been studied (reviewed in reference 43). This approach appears to reduce the incidence of chlamydia and syphilis but, rather expectedly, does not diminish rates of gonorrhea acquisition, given widespread resistance of gonococci to this agent. Dysbiosis, the development of antimicrobial resistance among other (commensal) organisms, and drug side effects are potential concerns associated with long-term antibiotic prophylaxis. Therefore, pathogen-targeting preventive strategies that are safe, cover a wide array of clinically relevant strains, and provide durable protection at a low cost are preferable.

Because of its broad bactericidal activity against a wide array of gonococcal isolates (26, 28, 44) and *in vivo* potency (31), MAb 2C7 is a promising candidate for the development of an antibody-based immunotherapeutic. A variant of MAb 2C7 designed to enhance complement activation improved its efficacy both *in vitro* and in a mouse model (31). While 2C7 with increased activity represents a significant advance in the development of an antigonococcal immunotherapeutic, the relatively short half-life of IgG may confine its use to the treatment of acute infections and may make it unsuitable for the ensuing months when repeat infections commonly occur. Fc mutations that increase binding to the neonatal Fc receptor (FcRn) have been successfully employed to increase the half-life of IgG MAbs (reviewed in reference 45). As an example, the complement C5 inhibitor ravulizumab has a 4-fold-longer half-life than the parent molecule eculizumab but still requires readministration to maintain sufficient serum levels (46, 47). Furthermore, the cost associated with MAb production and distribution may be an impediment to its use in low- and middle-income countries (LMICs), which bear a disproportionate burden of gonorrhea (18). In such settings, readministration of a MAb to prevent recurrent infections may not be cost-effective or practical. More affordable strategies to deliver antibody-based biologics with prolonged *in vivo* functionality are of great interest.

The DMAb platform has shown promise through the delivery of next-generation biologics with enhanced molecular potency that, importantly, demonstrate extended *in vivo* kinetics relative to their recombinant counterparts (38, 40). *In vivo* delivery of monoclonal antibodies engineered to augment specific innate effector mechanisms such as complement activation represents a novel application of this platform. Here, we designed and evaluated Fc-modified versions of MAb 2C7 using DNA-encoded, genetically optimized constructs. We first studied the *in vivo* expression kinetics and relative *in vitro* and *in vivo* bactericidal activities of these molecules. A single injection of optimized plasmids resulted in 100% seroconversion within 72 h. Expression levels continued to rise, peaking approximately 14 to 21 days postadministration before gradually contracting down to half of the maximal titer approximately 2 months postdelivery. These extended kinetics constitute a significant improvement in *in vivo* durability compared to biologic MAbs, which have a standard half-life of 14 to 21 days (48). As expected, all *in vivo*-produced 2C7 variants demonstrated comparable *in vitro* binding to LOS, but only the complement-active versions were able to facilitate *in vitro* killing of N. gonorrhoeae. Passive transfer of purified, *in vivo*-produced 2C7 DMAbs demonstrated the improved efficacy of complement-enhancing variants at dose-sparing levels relative to the parental wild-type DMAb. This was evident for the 2C7_E430G and 2C7_E345K variants, which, unlike the parental 2C7_WT DMAb, were efficacious *in vivo* at a single dose of 1 μg relative to control groups. Efficacy of the 2C7_E430G variant paralleled that with the recombinant molecule produced in CHO cells, as reported previously (31). Having established the expression kinetics, specificity, and antibacterial activity of these DMAbs, we next evaluated the ability of DNA-delivered 2C7 variants to facilitate clearance of N. gonorrhoeae infection at time points corresponding to acute (D8) and delayed (D65) infection. Not only did all complement-engaging 2C7 DMAbs provide enhanced clearance of primary infection, but they also demonstrated sustained *in vivo* functionality and efficacy against subsequent rechallenges administered 9 weeks after DMAb delivery. This represents a significant advantage of the DMAb platform over conventional MAbs.

The ability of DNA technology to facilitate the *in vivo* expression of complement-activating, functionally enhanced monoclonal antibodies over a prolonged period is an important advancement. The improved molecular functionality combined with additional optimization strategies previously shown to improve *in vivo* expression has resulted in rapid, robust, and stable production of a potent immunotherapeutic that hastens N. gonorrhoeae clearance following mucosal challenge for over 2 months following a single dose. Our data support the utility of complement-enhancing approaches to deliver advanced DNA-based immunotherapeutics that behave similarly to their recombinant counterparts, with a more durable profile and the potential to positively impact the treatment and prevention of N. gonorrhoeae. This approach also has broader implications for combatting other antibiotic-resistant infections that threaten global health.

## MATERIALS AND METHODS

### Ethics statement.

Use of animals was performed in strict accordance with recommendations in the *Guide for the Care and Use of Laboratory Animals* of the National Institutes of Health (49). Protocols were approved by the Institutional Animal Care and Use Committees (IACUCs) at the University of Massachusetts Medical School (protocol number A-1717) and The Wistar Institute (protocol 112779).

### Engineering of DMAbs.

The murine variable light chain (V_L_) and heavy chain (V_H_) nucleotide sequences from the hybridoma clone 2C7 were engineered at the DNA and RNA levels to enhance *in vivo* expression as previously described (35). The codon-optimized sequences, followed by those encoding human IgG1 CH1, CH2, CH3, and CL frameworks, were inserted into a modified pVax1 plasmid DNA expression vector under the control of the human cytomegalovirus (hCMV) promoter, optimized IgG leader sequences, and a bovine grown hormone (BGH) poly(A) signal; these components promote DMAb transcription, translation, and systemic distribution *in vivo*. A dual-plasmid system was used, generating a light-chain (LC) plasmid and a separate heavy-chain (HC) plasmid (2C7_WT) with the native, unmodified Fc human IgG1. In addition to the naturally complement-engaging 2C7 WT antibody, three additional HC variants were designed containing targeted mutations in the human IgG1 Fc domain that are known to modulate complement activation: (i) a complement-enhancing variant containing an E-to-G mutation at the 430 position in the CH_3_ domain (2C7_E430G), (ii) a second complement-enhancing variant containing an E-to-K modification at residue 345 in the CH_3_ domain (2C7_E345K), and (iii) a double mutant with a K-to-A mutation at residue 322 and a D-to-A modification at residue 270 (2C7_A/A), both located within the CH_2_ domain, which abrogate complement activation. In passive transfer challenge studies, a previously characterized anti-HIV DMAb was administered as an additional control for specificity (39).

### *In vitro* expression of 2C7 DMAbs.

2C7 variants were expressed *in vitro* using a mammalian cell line (Expi293 expression system; Gibco). Duplicate wells were transfected with equal amounts of HC and LC plasmid combinations (1 μg each/2.6 × 10^6^ cells) encoding (i) 2C7_WT, (ii) 2C7_E345K, (iii) 2C7_E430G, or (iv) 2C7_A/A DMAbs according to the manufacturer’s protocol. Following a 96-h incubation at 37°C, culture supernatants were harvested and analyzed for the presence of secreted 2C7 DMAbs via Western blotting and enzyme-linked immunosorbent assay (ELISA).

### Western blotting (*in vitro* expression).

To assess the expression and secretion of 2C7 DMAb variants, transfection supernatants were prepared under reduced conditions (containing 1× NuPage sample-reducing agent; Invitrogen), separated by electrophoresis on a NuPage 4 to 12% bis-Tris protein gel (Invitrogen), transferred to polyvinylidene difluoride (PVDF) membranes (Millipore membrane; Invitrogen iBlot2 dry blotting system), and blocked with for 1 h (Odyssey blocking buffer; LI-COR). Membranes were probed with anti-GAPDH antibody (Abcam; 1:5,000) for 2 h at room temperature (RT). Chimeric 2C7 DMAbs were detected with goat-anti-human IgG (LI-COR), and rabbit-anti-GAPDH (Invitrogen) was visualized with goat-anti-rabbit IgG (LI-COR), each diluted at 1:15,000, and incubated for 1 h at RT. Membranes were washed between antibody incubations with 1× phosphate-buffered saline (PBS) with 0.05% Tween 20 and imaged using an Odyssey CLX imager (LI-COR).

### Quantification and binding of 2C7 DMAbs.

2C7 DMAbs levels in culture supernatant and/or sera were determined via ELISA using 96-well, high-binding immunosorbent plates coated with 5 μg/ml of purified anti-human IgG-Fc (Bethyl Laboratories) overnight at 4°C. Wells were blocked (5% nonfat dry milk [NFDM]–1× PBS for 1 h at 23°C) and washed with 1× PBS–0.05% Tween 20 between subsequent incubations. Samples were serially diluted in 1× PBS–0.05% Tween–1% fetal bovine serum (FBS), plated in duplicate, and incubated for 2 h at RT. Purified human IgG(λ) (Bethyl Laboratories) was used to create a standard curve. Bound antibodies were detected with anti-human light-chain (λ) antibody conjugated to horseradish peroxidase (Bethyl Laboratories) diluted 1:10,000 for 1 h at RT. Plates were developed with SigmaFast *o*-phenylenediamine dihydrochloride (OPD) substrate (Sigma) for 10 min and stopped with 2 N H_2_SO_4_. Plates were read using a BioTek Synergy 2 plate reader (Biotek) at the 450-nm wavelength. To test LOS binding of 2C7, DMAbs were quantified by ELISA using plates coated with purified LOS containing the 2C7 epitope (N. gonorrhoeae strain 15253) as previously described (27). Microtiter wells were coated with LOS (80 μg/ml in PBS) for 15 h at 4°C. Wells washed twice with PBS–0.05% Tween 20 and then blocked with the same buffer for 1 h. Dilutions of mouse sera were added to wells and incubated for 1 h at 37°C. Bound anti-2C7 human IgG1 chimeric Ab was detected with goat anti-human IgG-Fc conjugated to alkaline phosphatase (AP; Sigma) at a 1:1,000 dilution in PBS–0.05% Tween 20 and detected with *p*-nitrophenyl phosphate substrate. A standard curve was generated using chimeric human IgG1 2C7.

### Mouse strains.

Female 6- to 8-week-old Jh mice (BALB/c background; strain 1147-F), which contain a targeted deletion in the V_J_ segment that prohibits the natural production of antibodies against the *in vivo*-produced DMAb constructs, were purchased from Taconic. Female 6- to 8-week-old BALB/c nude mice (strain CAnN.Cg-Foxn1nu/Crl), which lack a thymus and therefore contain no T cells, were purchased from Charles River. Protection experiments were performed using JHD mice (which have targeted deletions of JH gene segments and also are Ab deficient) in a BALB/c background (provided by Ann Rothstein, University of Massachusetts Medical School).

### *In vivo* DMAb administration and serum collection.

Nude, Jh, and JHD mice were injected with 100 μg total plasmid DNA (50 μg LC plus 50 μg HC) encoding 2C7_WT, 2C7_E345K, 2C7_E430G, or 2C7_A/A. Additional control groups received empty pVax plasmid (39). Plasmids were formulated in water containing hyaluronidase (12 U/site; Sigma) and injected intramuscularly (i.m.) in the tibialis anterior (TA) muscle followed by electroporation at the site using the CELLECTRA 3P adaptive constant-current device (Inovio Pharmaceuticals), which administers transient and mild pulses to facilitate DNA uptake. Blood was collected at weekly intervals following administration to monitor *in vivo* expression kinetics and functional potency against vaginal colonization of N. gonorrhoeae.

### Human complement.

Complement-active normal human serum that was depleted of IgG and IgM was obtained from Pel-Freez Biologicals (catalog no. 34010-1).

### Serum bactericidal assay.

Serum bactericidal assays were performed as described previously (29). Bacteria harvested from overnight cultures were repassaged on chocolate agar, grown for 6 h, and suspended in Hanks balanced saline solution (HBSS) containing 0.15 mM CaCl_2_ and 1 mM MgCl_2_ (HBSS^++^). Approximately 2,000 CFU of suspended bacteria were incubated with NHS and MAb 2C7 (concentrations are specified for each experiment). The final reaction volumes were maintained at 150 μl. Aliquots of 25 μl of reaction mixtures were plated onto chocolate agar in duplicate at the beginning of the assay (*t*_0_) and again after incubation at 37°C for 30 min (*t*_30_). Survival was calculated as the number of viable colonies at *t*_30_ relative to the number at *t*_0_.

### Mouse vaginal colonization model of gonorrhea.

Female mice (either wild-type BALB/c or JHD) 6 to 8 weeks of age (The Jackson Laboratory) in the diestrus phase of the estrous cycle were started on treatment (that day) with 0.5 mg Premarin (Pfizer) in 200 μl water given subcutaneously on each of 3 days; −2, 0, and +2 days (i.e., before, the day of, and after inoculation) to prolong the estrus phase of the cycle and promote susceptibility to N. gonorrhoeae infection (50). Premarin is a mixture of sodium estrone sulfate and sodium equilin sulfate and, as concomitant components, sodium sulfate conjugates of 17α-dihydroequilin, 17α-estradiol, and 17β-dihydroequilin. Antibiotics (vancomycin, colistin, neomycin, trimethoprim, and streptomycin) that are ineffective against N. gonorrhoeae were used to reduce competitive microflora (50). Mice were challenged intravaginally with the inoculum indicated in the figure legend for each experiment of N. gonorrhoeae strain FA1090 (piliated [Pil^+^] and expressing the opacity protein [Opa]) as previously described (51). Infection was monitored daily through vaginal swabbing and bacterial enumeration (CFU).

### DMAb purification and passive transfer.

Serum prepared from blood obtained through cardiac puncture of 21-day-old JHD mice given pVax encoding either the control (nonspecific) antibody or one of the 2C7 MAbs was passaged over protein A/G agarose. Bound IgG was eluted with 0.1 M glycine (pH 3.0) and immediately neutralized with 0.5 M Tris (pH 8.0). Buffer was exchanged to PBS by spin-concentration dialysis using Amicon Ultra centrifugal filters with a 30-kDa cutoff. IgG was sterilized through a 0.22-μm filter, and protein concentration was determined by absorbance at 280 nm and the Pierce BCA protein assay kit prior to use in mice. For passive transfer challenge experiments, serum-derived purified 2C7 variants were delivered via intravenous injection at 5 μg or 1 μg. The following day, mice were challenged intravaginally, and infection was monitored as described above.

### Dosing studies of 2C7_E430G recombinant IgG.

Recombinant chimeric 2C7_E430G IgG, previously characterized for its improved complement activation and enhanced *in vivo* potency (31), was produced at Genmab using traditional bioprocessing approaches. This IgG was intravenously injected into naive mice at dose of 25, 5 or 1 μg. The following day, mice were challenged as described above.

### Statistical analyses.

In cases in which the means between multiple groups were compared (*in vivo* DMAb titers, *in vitro* bacterial killing, and AUC values), Kruskal-Wallis nonparametric rank-sum tests were used due to small group size and/or contained skewed/kurtotic distributions. Pairwise comparisons were conducted using Dunn’s *post hoc* test. A nonparametric Spearman correlation was performed to assess the relationship between DMAb serum titers and *in vitro* bactericidal activity. For challenge experiments, we used the various independent groups of DMAb-treated mice to estimate and test three characteristics of the data (29, 52): time to clearance, longitudinal trends in mean log_10_ CFU, and the cumulative CFU as AUC. Longitudinal trends of log_10_ CFU over time were analyzed using a mixed-effects model with a cubic function over time. Median time to clearance was estimated using Kaplan-Meier survival curves; times to clearance were compared between groups using the Mantel-Cox log-rank test. Significance was set using Bonferroni’s correction when more than two groups were compared and is indicated for each experiment. The mean AUC (log_10_ CFU versus time) was computed for each mouse to estimate the bacterial burden over time (cumulative infection); the means under the curves were compared between groups using the Kruskal-Wallis test and Dunn’s *post hoc* test. *Post hoc* power calculations for pairwise comparisons between groups of mice in Fig. 4 and 5 are shown in Table S1.

10.1128/mBio.00242-21.7 TABLE S1 Power analysis of pairwise comparison of AUCs (two-tailed test) for Fig. 4 and 5. Download Table S1, PDF file, 0.04 MB. Copyright © 2021 Parzych et al. 2021 Parzych et al. https://creativecommons.org/licenses/by/4.0/ This content is distributed under the terms of the Creative Commons Attribution 4.0 International license.
