# Supplementary figures and images for: Synthetic DNA Delivery of an Optimized and Engineered Monoclonal Antibody Provides Rapid and Prolonged Protection against Experimental Gonococcal Infection
Source: mBio. 2021 Mar 16;12(2):e00242-21. doi: 10.1128/mBio.00242-21 (PMC8092225; doi:10.1128/mBio.00242-21)

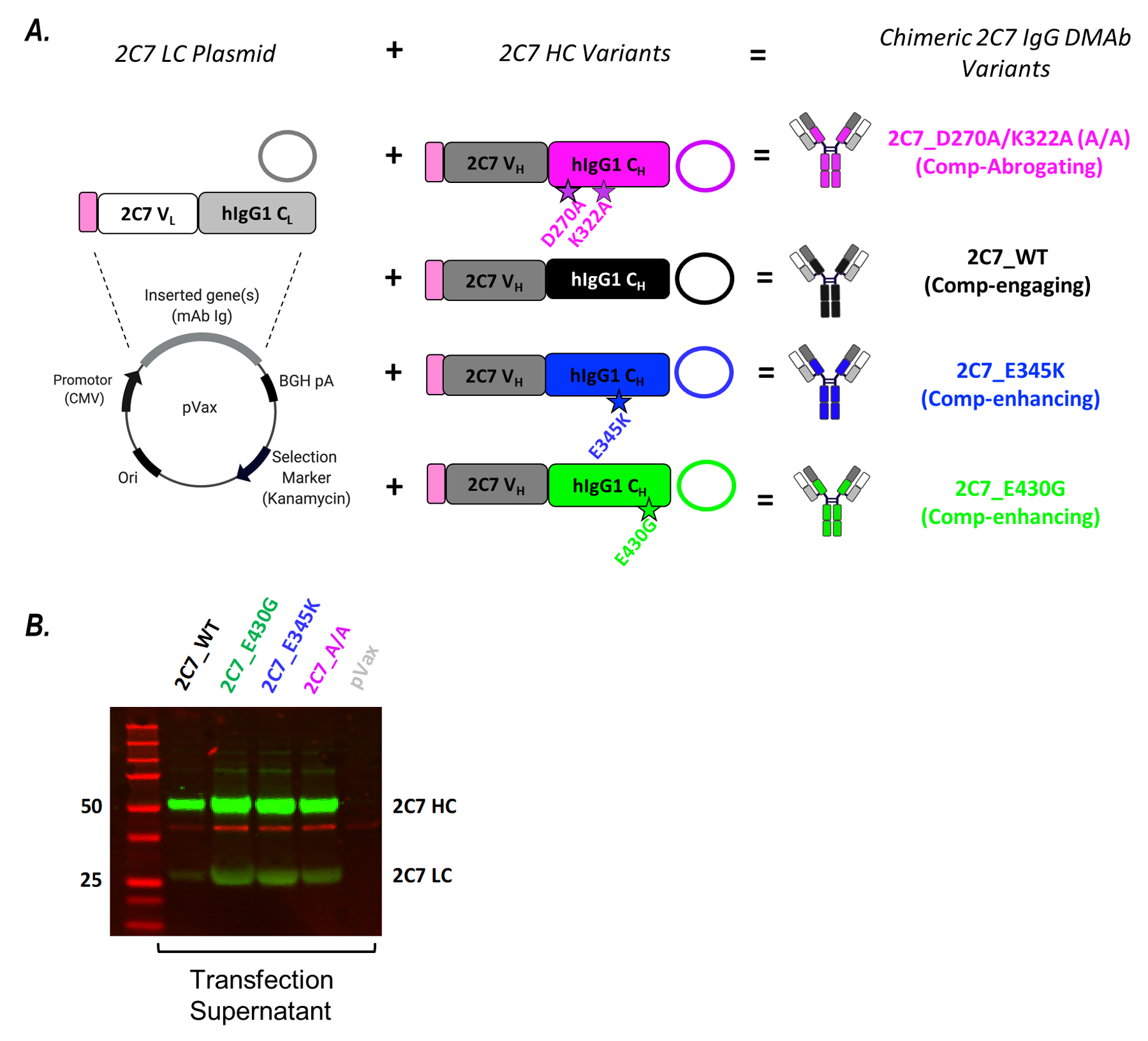

Supplement: FIG S1 [file mBio.00242-21-sf001.tif]

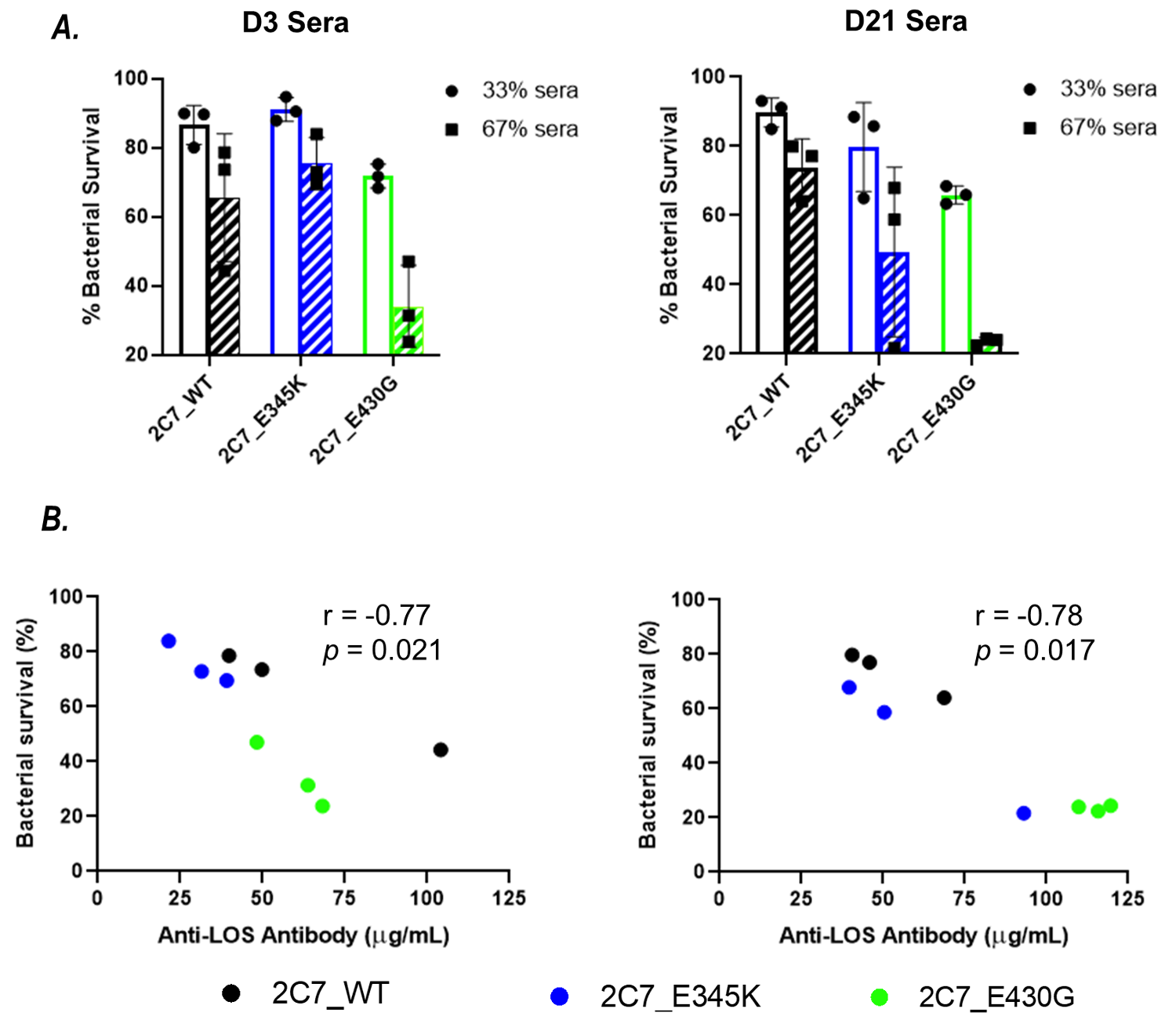

Supplement: FIG S2 [file mBio.00242-21-sf002.tif]

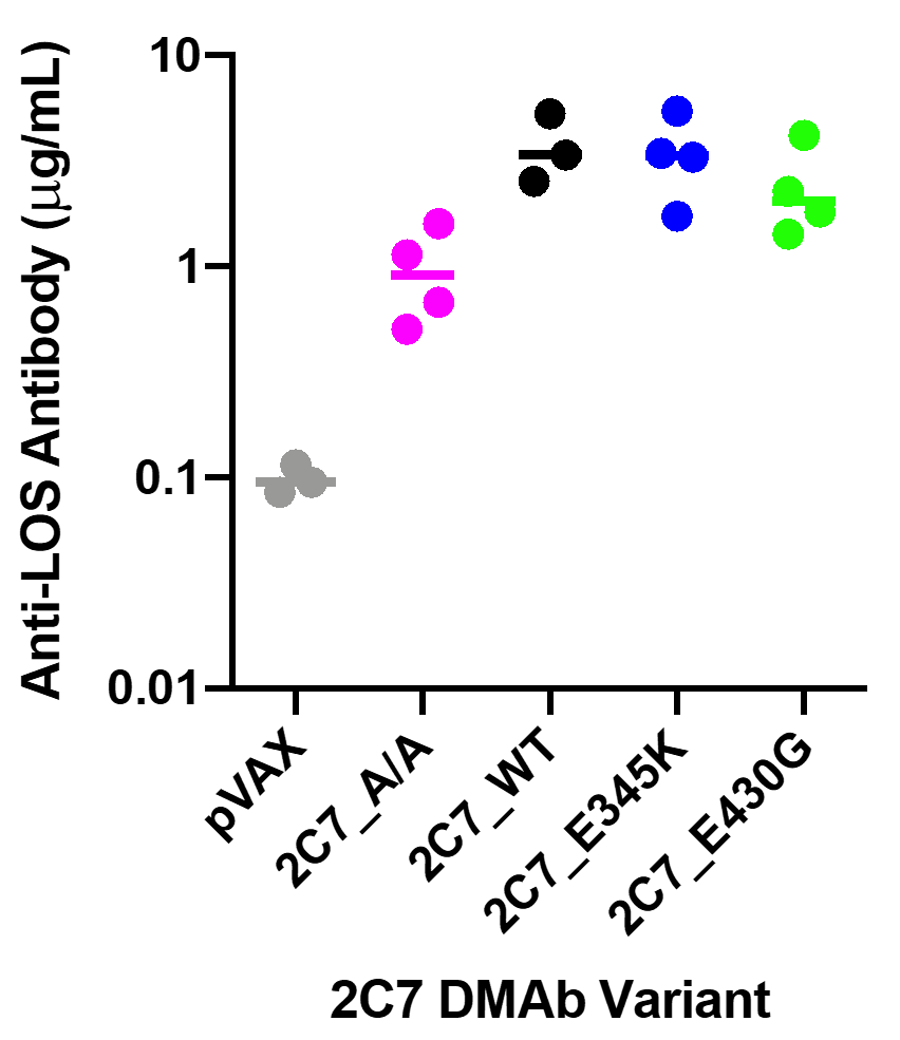

Supplement: FIG S3 [file mBio.00242-21-sf003.tif]

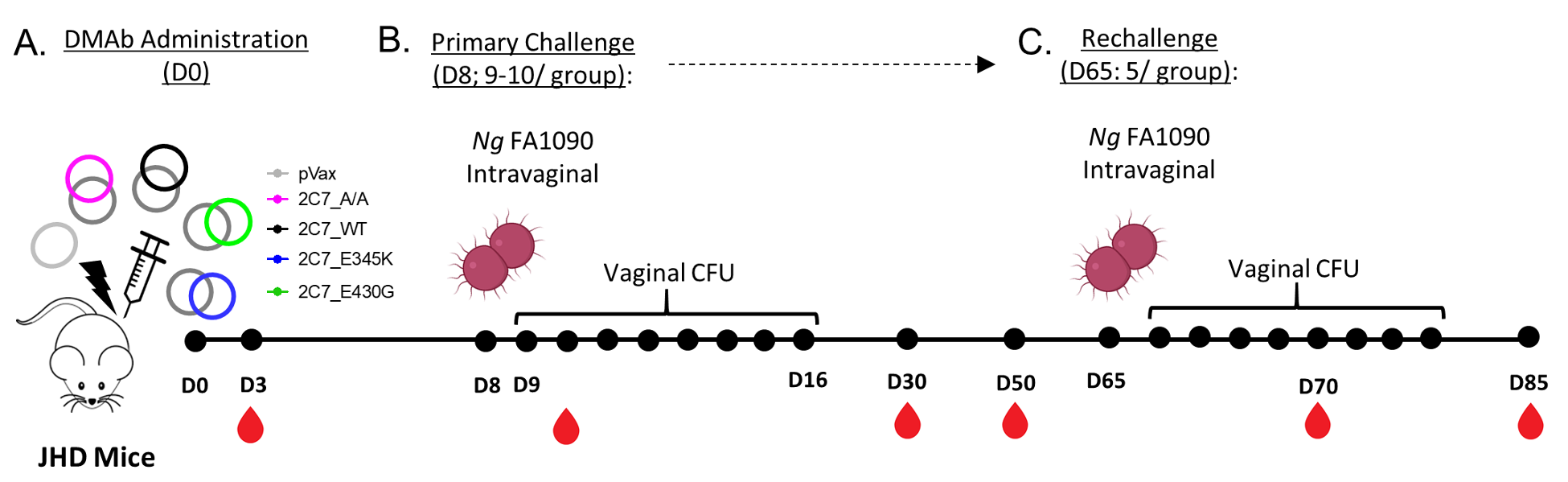

Supplement: FIG S4 [file mBio.00242-21-sf004.tif]

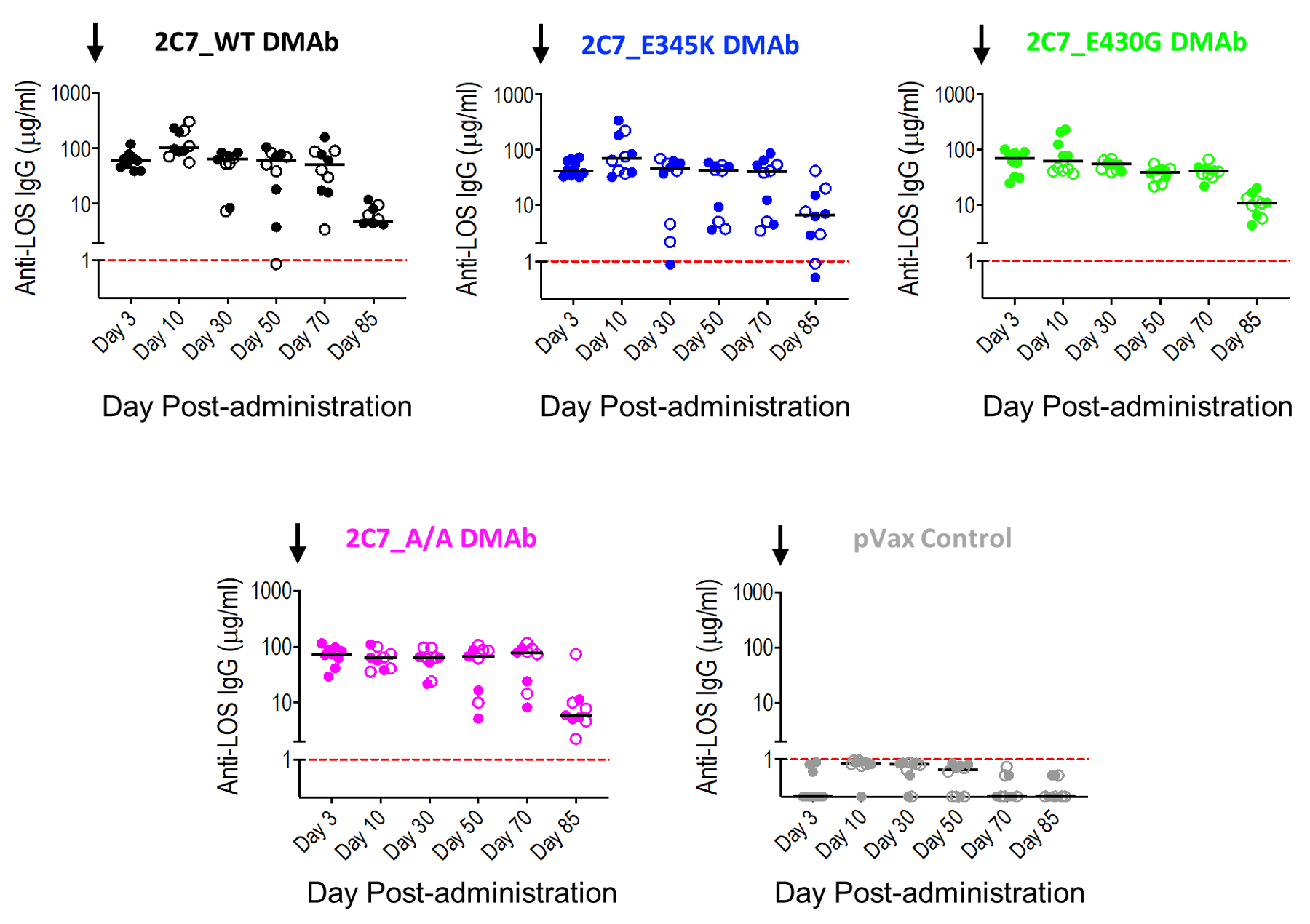

Supplement: FIG S5 [file mBio.00242-21-sf005.tif]

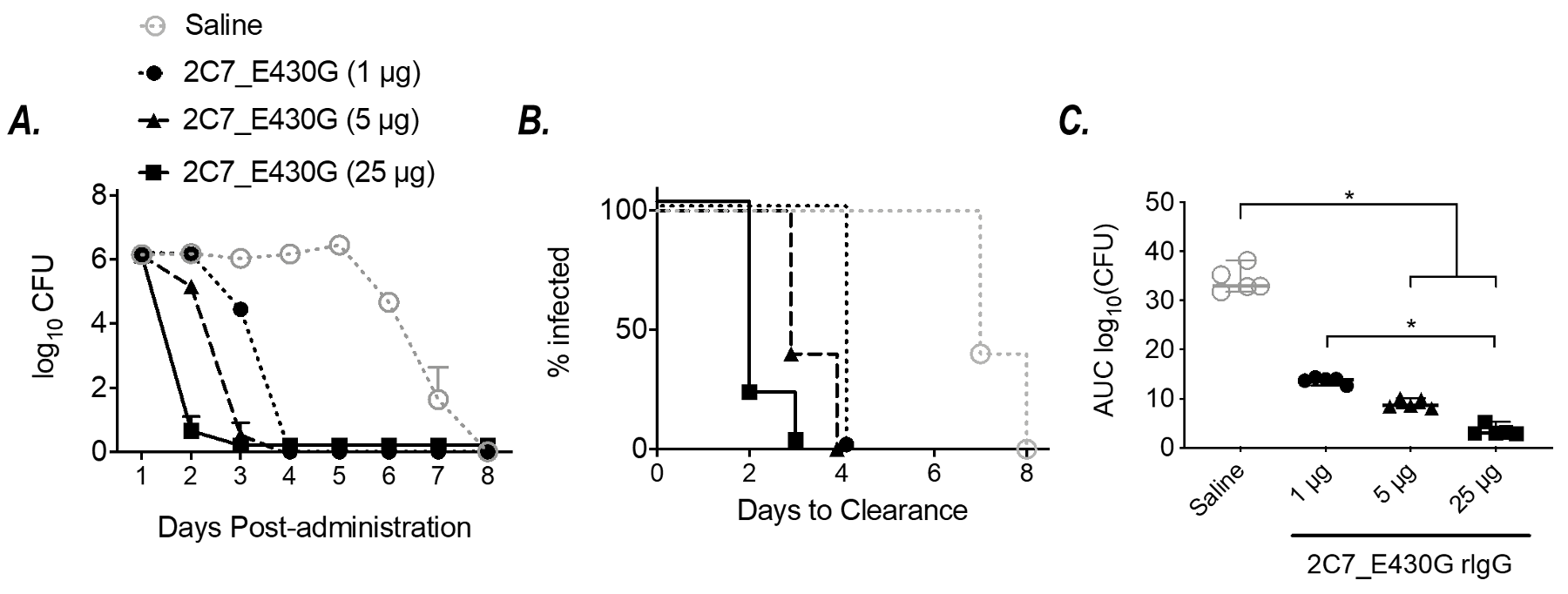

Supplement: FIG S6 [file mBio.00242-21-sf006.tif]
