# Supplementary material for: Synthetic DNA Delivery of an Optimized and Engineered Monoclonal Antibody Provides Rapid and Prolonged Protection against Experimental Gonococcal Infection
Source: mBio. 2021 Mar 16;12(2):e00242-21. doi: 10.1128/mBio.00242-21 (PMC8092225; doi:10.1128/mBio.00242-21)
Supplement: TABLE S1 [file mBio.00242-21-st001.pdf]

Table S1. Power analysis of pairwise comparison of AUCs (two-tailed test) for Fig 4 and Fig 5[A]

Comparison	Mean (SD)	Statistical Power (%)
Figure 4 (n=5/group)		
pVAX vs 2C7_E345K	38.2 (3.9) vs 22.5 (11.7)	81.5
pVAX vs 2C7_E430G	38.2 (3.9) vs 8.1 (5.4)	100
2C7_A/A vs 2C7_E430G	36.1 (4.3) vs 8.1 (5.4)	100
2C7_WT vs 2C7_E430G	29.5 (6.0) vs 8.1 (5.4)	100
2C7_A/A vs 2C7_E345K	36.1 (4.3) vs 22.5 (11.7)	68.7 (P>0.05, ns)
Figure 5D (n=5/group)		
**WT BALB/c mice (Fig 5D, left graph)**		
Saline vs 2C7_WT (5 µg)	32.79 (1.8) vs 9.3 (0.4)	100
Saline vs 2C7_E345K (5 µg)	32.79 (1.8) vs 7.9 (0.3)	100
Saline vs 2C7_E430G (5 µg)	32.79 (1.8) vs 6.1 (0.6)	100
Saline vs 2C7_E345K (1 µg)	32.79 (1.8) vs 12.7 (0.5)	100
Saline vs 2C7_E430G (1 µg)	32.79 (1.8) vs 7.8 (0.5)	100
Non-sp. DMAb vs 2C7_WT (5 µg)	35.2 (0.9) vs 9.3 (0.4)	100
Non-sp. DMAb vs 2C7_E345K (5 µg)	35.2 (0.9) vs 7.9 (0.3)	100
Non-sp. DMAb vs 2C7_E430G (5 µg)	35.2 (0.9) vs 6.1 (0.6)	100
Non-sp. DMAb vs 2C7_E345K (1 µg)	35.2 (0.9) vs 12.7 (0.5)	100
Non-sp. DMAb vs 2C7_E430G (1 µg)	35.2 (0.9) vs 7.8 (0.5)	100
**JHD mice (Fig 5D, right graph)**		
Saline vs 2C7_WT (5 µg)	37.3 (3.0) vs 9.8 (1.3)	100
Saline vs 2C7_E345K (5 µg)	37.3 (3.0) vs 8.0 (0.6)	100
Saline vs 2C7_E430G (5 µg)	37.3 (3.0) vs 6.6 (0.4)	100
Saline vs 2C7_E345K (1 µg)	37.3 (3.0) vs 13.3 (0.6)	100
Saline vs 2C7_E430G (1 µg)	37.3 (3.0) vs 9.0 (1.1)	100
Non-sp. DMAb vs 2C7_WT (5 µg)	32.9 (1.4) vs 9.8 (1.3)	100
Non-sp. DMAb vs 2C7_E345K (5 µg)	32.9 (1.4) vs 8.0 (0.6)	100
Non-sp. DMAb vs 2C7_E430G (5 µg)	32.9 (1.4) vs 6.6 (0.4)	100
Non-sp. DMAb vs 2C7_E345K (1 µg)	32.9 (1.4) vs 13.3 (0.6)	100
Non-sp. DMAb vs 2C7_E430G (1 µg)	32.9 (1.4) vs 9.0 (1.1)	100

[A] power calculation performed using: https://www.sphanalytics.com/statistical-power-calculator-using-average-values/
